# Exploring an Aptamer-Based Approach to Assess Canine Parvovirus Integrity After Disinfection Treatment

**DOI:** 10.3390/v17101309

**Published:** 2025-09-27

**Authors:** Md Anik Ashfaq Khan, Ahmed Abd El Wahed, Stefan Breuers, Knut Krohn, Günter Mayer, Torsten Schöneberg, Uwe Truyen

**Affiliations:** 1 Institute of Animal Hygiene and Veterinary Public Health, University of Leipzig, An den Tierkliniken 1, 04103 Leipzig, Germany; anik.ashfaq@gmail.com (M.A.A.K.);; 2 Life and Medical Sciences Institute (LIMES), University of Bonn, Carl-Troll-Straße 31, 53115 Bonn, Germany; 3 Center of Aptamer Research & Development, University of Bonn, Gerhard-Domagk-Straße 1, 53121 Bonn, Germany; 4 Core Unit DNA Technologies, University of Leipzig, Philipp-Rosenthal-Str. 55, 04103 Leipzig, Germany; 5 Rudolf Schönheimer Institute for Biochemistry, University of Leipzig, Johannisallee 30, 04103 Leipzig, Germany

**Keywords:** canine parvovirus, disinfection, aptamer

## Abstract

Virus inactivation exhibits varying disinfection kinetics due to structural or genomic differences. Standard post-disinfection assessment relies on observing cytopathic effects in inoculated cell cultures, which are limited by sensitivity, availability, cost, and turnaround time. This study explores nucleic acid aptamers as molecular sensors to differentiate between intact and post-disinfection virus particles. To discover aptamers, 12 cycles of an automated SELEX (Systematic Evolution of Ligands by Exponential Enrichment) experiment were performed using recombinant (r)-VP2 protein of canine parvovirus (CPV). Enrichment of single stranded (ss) DNA binders was evaluated by sequencing the enriched libraries. The most abundant sequences were tested for binding with coated rVP2 and CPV (intact and treated with heat and peracetic acid (PAA) disinfectant) followed by detection using PCR. Binding specificity was assessed using intact and heat-treated feline panleukopenia virus (FPV) and porcine parvovirus (PPV). Sequencing of the DNA libraries from selection cycle 6 and cycle 12 products showed individual sequence enrichment with maximum frequencies of 2.14% and 8.65%, respectively. The top three abundant sequences from each cycle confirmed rVP2 binding. In the case of CPV, only heat-treated and PAA-treated CPV showed binding to the candidate sequences. However, reduced binding to the CPV-specific antibody was observed for rVP2 and treated CPV compared to intact CPV. No apparent binding of the tested sequences was observed for FPV and PPV. Aptamers binding to denatured but not intact CPV demonstrate the potential to distinguish between the two states, providing a basis for developing a molecular assay to assess disinfection efficacy.

## 1. Introduction

An outbreak of infectious disease demands a rapid response to control its spread. Amid the challenges of developing therapeutics and/or vaccines in a short time, especially for fast-spreading viral diseases such as COVID-19 [1] and endemic viruses, it is crucial to use the appropriate disinfection material to limit infection transmission. Hence, virucidal disinfectants have gained increasing interest due to their broad applications.

To formulate standardized test protocols for determining disinfectant efficacy against emerging pathogens, European standards, as well as the German Veterinary Medical Society (DVG) guidelines for assessment, recommend the use of relevant surrogate viruses with similar biophysical properties. The choice of surrogate viruses is critical for establishing a reliable standardized test [2,3,4]. Furthermore, standard disinfectant testing protocols involve determining virucidal activity through suspension and solid carrier tests, which require well-established cell culture methods to calculate viral infectivity titers before and after treatment. However, the efficacy of viral inactivation varies significantly, largely due to structural differences among viruses. For instance, enveloped viruses are more susceptible to chemical disinfectants such as alcohols (ethanol, isopropanol) and quaternary ammonium compounds (e.g., benzalkonium chloride) that contain lipid bilayer-disrupting active substances than non-enveloped viruses, which exhibit stronger hydrophilic properties [4,5]. While the representation of surrogates for enveloped viruses is relatively straightforward due to their higher susceptibility to chemical disinfectants, non-enveloped viruses, such as *Parvoviridae*, generally exhibit lower susceptibility, with significant variability even within the same family or genus. Their susceptibility to virucidal agents is also highly dependent on the chemical composition of the disinfectant [6,7]. For example, aldehyde- or chlorine-based agents (e.g., sodium hypochlorite, glutaraldehyde, formaldehyde), and oxidizing agents (e.g., hydrogen peroxide, chlorine dioxide, peracetic acid) are often the inactivating agents used for both non-enveloped and enveloped viruses. The choice and application of a chemical disinfectant are also dependent on mechanism of action, stability and safety. For instance, peracetic acid (PAA), which is a highly oxidizing and volatile agent exerts its activity by modifying amino acid side chains and disrupting structural stability through alterations of surface properties and essential non-covalent interactions [8]. Unlike corrosive disinfectants such as chlorine-based agents, PAA decomposes into non-toxic byproducts in aqueous solutions, and it has been demonstrated to efficiently inactivate parvoviruses with established use in veterinary and environmental disinfection [9,10,11].

Nevertheless, pathogen-specific evaluations of virucidal efficacy are more suitable for understanding a test reagent’s effectiveness. However, the standard cell-culture-based cytopathic effect remains the primary method for determining and quantifying viral infectivity. This propagation-dependent strategy poses challenges for virucidal efficacy tests, such as the requirement for higher biosafety levels (BSL), unavailability of virus collections and cell culture methods for emerging pathogens, residual disinfectant effects in cell cultures, cytotoxic disinfectant components and the high cost and extended turnaround time of protocols. While molecular tests such as PCR have shown promise in detecting viral infectivity in clinical samples to some extent [12,13], the effectiveness of PCR for determining infectivity following disinfection is limited [14], possibly due to residual disinfectant effects and surface contaminants that interfere with nucleic acids and PCR enzymes. Furthermore, genomic content detection via PCR can be non-specific, as it may not distinguish between infectious and non-infectious particles [15]. On the other hand, distinguishing between intact virions and denatured particles in terms of infectivity provides a reliable benchmark for assessing virucidal performance, thereby making it an essential component of disinfectant quality control [16,17]. In this context, assays that selectively detect and quantify intact virions amidst degraded particles can serve as a primary evaluation step for disinfectant performance.

Few reports exist on alternative methods that can differentiate between intact infectious viruses and non-infectious forms rendered by chemical disinfection. In this context, since most chemical disinfectants first interact with and destabilize the viral capsid, nucleic acid aptamers could leverage the conformational differences between these states on the virus surface. Aptamers are short, single-stranded (ss) RNA or DNA molecules with specific nucleotide sequences that fold into unique tertiary structures and bind specifically to target molecules, which range from biological macromolecules like proteins and peptides to small molecules like metal ions. Like antibodies, aptamers can exhibit high affinity and specificity for their targets. However, unlike antibodies, aptamers are synthesized chemically rather than biologically, making them more cost-effective, quicker to produce, and more stable across a range of temperatures, pH levels, and ionic conditions. They also exhibit fewer batch-to-batch variations and are scalable. The ability of nucleic acid aptamers to bind viral envelope proteins has long been demonstrated, along with their diagnostic and therapeutic applications [18,19].

In this study, aptamers were assessed for their potential as molecular sensors to differentiate intact canine parvovirus (CPV) particles from their denatured forms induced by heat and chemical treatment. For this purpose, recombinant VP2 protein, produced in a bacterial host, was used to discover new ssDNA aptamers via the Systematic Evolution of Ligands by EXponential Enrichment (SELEX) method, which is central to aptamer research. SELEX begins by incubating an ssDNA or RNA library with a target molecule, followed by recovery of bound sequences and amplification via PCR to create a pool of potential aptamer sequences for the next selection round. Typically, five to twenty rounds are required to enrich the selective nucleic acid pool and identify candidate target-binding aptamers [20].

## 2. Materials and Methods

### 2.1. Protein and Virus Preparation

To select aptamers against the capsid of CPV, His-tagged recombinant (r)-VP2 protein (Uniprot ID: P61826, partial, 30–553 amino acids) of type 2 CPV was expressed in *E. coli*, followed by nickel column affinity purification (Cusabio, Houston, TX, USA). Upon receiving lyophilized content, the protein was reconstituted with deionized water (1 µg protein/µL H_2_O) and checked for purity by SDS-PAGE for immediate use or stored at −80 °C with 50% glycerol as per the manufacturer’s recommendation. To validate rVP2 aptamer binding to the intact virus, CPV type-2 (vBI265) was propagated on Crandell-Rees Feline Kidney (CRFK) cells in Dulbecco’s medium (DMEM: 3.7 g/L NaHCO_3_, 4.5 g/L d-glucose, 0.5 g/L L-glutamine) supplemented with 5% fetal bovine serum (FBS, PAA Laboratories, Pasching, Austria), 1% Penicillin-Streptomycin, and 1% non-essential amino acids. The propagation was conducted at 37 °C in 5% CO_2_ for 6–7 days. Virus-containing supernatants were clarified by two successive centrifugations at 3200× *g* at 4 °C for 10 min, and pellets discarded after each round. Virus titer as 50% tissue culture infectious dose (TCID_50_) per milliliter was measured as published before [21] and stored at −20 °C. Similar culture and storage conditions were maintained for feline panleukopenia virus (FPV) strain vBI292. The vBI265 and vBI292 were kindly provided by Colin Parrish. The porcine parvovirus (PPV) strain 143a was propagated on swine testis (ST) cells maintained in DMEM supplemented with 10% FBS and 1% Penicillin-Streptomycin and purified essentially as described for the carnivore parvoviruses. Because of their phylogenetic proximity to CPV, FPV and PPV strains were included to evaluate the specificity of aptamer candidates that bind to rVP2 of CPV.

### 2.2. SELEX

To identify ssDNA aptamers that bind to rVP2, an automated selection procedure was employed [22]. The preparation of protein and selection of aptamers were performed as per the published protocol [23] and summarized hereafter.

#### 2.2.1. Protein Immobilization

For immobilization of His-tagged rVP2 proteins, 214 pmol of rVP2 proteins prepared in 1 mL wash/binding buffer (50 mM sodium phosphate, pH 8.0, 300 mM NaCl, 0.01% Tween^®^-20) were coupled to 100 µL of Dynabeads bead solution (40 mg beads/mL) (ThermoFisher, Waltham, MA, USA), according to the manufacturer’s protocol. Bead-retaining buffer was discarded using a DynaMag™-2 magnetic pull (ThermoFisher, MA, USA) before coupling. The mixture was incubated gently for 30 min at 4 °C on an orbital shaker, followed by three consecutive washing steps with 1 mL wash/binding buffer. An additional washing step with storing buffer (1.25× PBS; 171.25 mM NaCl (Fisher Scientific, Hampton, NH, USA), 3.38 mM KCl (Roth), 12.5 mM Na_2_HPO_4_ (Roth, Karlsruhe, Germany), 2.2 mM KH_2_PO_4_ (Roth, Karlsruhe, Germany), pH 7.4; 1 mg/mL Albumin (BSA) Fraction V (pH 7.0) (AppliChem, Darmstadt, Germany); 3.25 mM MgCl_2_) was performed before resuspending rVP2-bound beads in 1 mL of storing buffer [23].

#### 2.2.2. ssDNA Library Preparation

For the selection of ssDNA aptamers, a DNA library (D2) in which each sequence of 40 random nucleotides is flanked by a constant primer binding sites (5′–GGG AGA GGA GGG AGA TAG ATA TCA A–N40–T TTC GTG GAT GCC ACA GGA C–3′) was used (Ella Biotech GmbH, Fürstenfeldbruck, Germany). For amplifying the library, the following primers were used: forward primer (D2fw) 5′–GGG AGA GGA GGG AGA TAG ATA TCA A–3′ and reverse primer (Phos-D2rv) 5′ P–GTC CTG TGG CAT CCA CGA AA–3′. The PCR amplification reaction was performed with colorless GoTaq^®^ Flexi Buffer (Promega, Madison, WI, USA), 2 mM MgCl_2_ (Roth, Karlsruhe, Germany), 0.2 mM dNTPs (Genaxxon, Ulm, Germany), 1 µM of D2fw and Phos-D2rv primers, and 1 µL GoTaq^®^ G2 Flexi DNA Polymerase (5 U/µL, Promega) in a total reaction volume of 100 µL under the cycling conditions of 30 s at 95 °C, 30 s at 62 °C, and 30 s at 72 °C for 8 cycles in a TRobot thermal cycler (Biometra, Jena, Germany) [23].

#### 2.2.3. Selection Procedure

The automated selection was initiated by pipetting 0.5 nmol of D2 ssDNA library (Biomek NXP, Beckman Coulter, Brea, CA, USA) onto the immobilized rVP2 protein, followed by incubation for 30 min at 37 °C with shaking at 700 rpm. Intermittent pipetting was performed every 5 min during incubation to ensure mixing. The selection buffer was composed of PBS/3 mM MgCl_2_/0.8 mg/mL BSA (PBS: 137 mM NaCl (Fisher Scientific, NH, USA), 2.7 mM KCl (Roth, Karlsruhe, Germany), 10 mM Na_2_HPO_4_ (Roth, Karlsruhe, Germany), and 1.76 mM KH_2_PO_4_ (Roth, Karlsruhe, Germany), pH 7.4). After incubation, the samples were washed twice with 100 µL wash buffer (PBS/3 mM MgCl_2_). Prior to PCR, the bound ssDNA molecules were eluted by incubation in ddH_2_O for 5 min at 80 °C. In the first four selection cycles, 18 PCR cycles were performed, and in all subsequent selection cycles, 16 PCR cycles were used. For all steps performed on the TRobot, an arched autosealing lid (Bio-Rad, Hercules, CA, USA) was utilized to seal the reaction plate. Following PCR, a lambda exonuclease digestion (final concentration 20 U, ThermoFisher) was carried out for 60 min at 37 °C to generate ssDNA for the next selection cycle, while an aliquot of dsDNA was stored to check the PCR product by agarose gel electrophoresis (AGE) (4% gel prepared in Tris-Borate EDTA buffer) visualized using the Genoplex system (VWR, Radnor, PA, USA) [23].

### 2.3. Evaluation of Enrichment of Nucleic Acid Binders

Enrichment of nucleic acid sequences was evaluated by next-generation sequencing (NGS) using the Illumina’s NovaSeq platform. For this, SELEX dsDNA products from the initial amplification (C1), mid-round (C6), and final round (C12) were amplified with modified D2 primers containing partial Illumina adapter at the 5′ end: 5′-TACACGACGCTCTTCCGATCT-D2fw and 5′-AGACGTGTGCTCTTCCGATCT-D2rv. Purification was performed using the NucleoSpin Gel and PCR Clean-up kit (Macherey-Nagel, Düren, Germany) following the manufacturer’s protocol. The selection product (R4) for streptavidin (unpublished) was sequenced as a background control of enriched sequences. After sequencing, primer and adapter sequences were trimmed, from the raw sequence data and low-quality reads were removed using fastp v0.20.0 [24]. Amplified sequences from the initial round (C1 and R1) served as negative controls for enrichment.

### 2.4. Binding Evaluation of Aptamer Candidates to rVP2 Protein and Cultured Virus

To evaluate the binding behavior of ssDNA aptamer candidates to rVP2 and cultured virus, ssDNA candidates with the highest occurrences in ranks C6 and C12 were synthesized (Tib-Molbiol, Berlin, Germany) and reconstituted with deionized water. Additionally, a simple plate-based method was formulated as summarized below.

#### 2.4.1. Obtaining Optimum Test rVP2 Concentration from CPV-Specific ELISA

To determine the optimum concentration of rVP2 for testing, the concentration of purified CPV was measured as TCID_50_/_mL_, and 100 µL of serially diluted CPV virus in PBS (pH 7.4) was coated onto a 96-well flat-bottom ELISA plate (Corning, NY, USA). An optimized antibody-ELISA assay was performed based on a previously published protocol [25]. Briefly, the plates were sealed to prevent evaporation and left at room temperature (RT) overnight. After washing three times with 100 µL of 1× wash buffer (PBS, 0.05% Tween-20), the wells were blocked with 100 µL of 5% skim milk (Roth, Karlsruhe, Germany) prepared in PBS and incubated for 2 h at RT. Following another three washes, 100 µL of a 1:50 dilution of monoclonal antibody mix [19] in PBS was added to each well and incubated for 1.5 h with gentle shaking at RT. After three additional washes, 100 µL of HRP-conjugated goat anti-mouse antibody (1:2500 dilution, Jackson, PA, USA) was added to each well and incubated for 30 min. The reaction was developed with 100 µL of 3,3′,5,5′-tetramethylbenzidine (TMB) substrate solution after the same washing steps, left for 15 min in the dark, followed by the addition of stop solution (1 N H_2_SO_4_). The absorbance at 450 nm was immediately read using a Labsystems Multiskan Ascent spectrophotometer (ThermoFisher, MA, USA).

#### 2.4.2. Assessment of rVP2-ssDNA Binding

In accordance, two concentrations of rVP2 (low: 100 nM, theoretically equivalent to 100 TCID_50_ virus particles, and high: 1.5 µM) were adjusted to 100 µL PBS (pH 7.4) and coated onto a 96-well flat-bottom ELISA plate, following the CPV-ELISA protocol stated above. However, instead of monoclonal antibodies, individual ssDNA suspensions were used. For this, reconstituted ssDNA were first incubated at 85 °C for 2 min to resolve any structural artifacts, followed by immediate cooling on ice for 1 min and then resting at RT for 10 min. The candidate aptamer binding solution was then prepared as 160 pmol ssDNA/3 mM MgCl_2_/50 µL PBS before adding to the well. Following 2 h of incubation at RT, the wells were washed three times, and 50 µL of Buffer DL nucleic acid extraction buffer (Xpedite, Bayern, Germany) was added to each well, sealed with a plate sealer, and incubated for 30 min at 70 °C. The total volume was transferred to a 1.5 mL tube before 12 cycles of PCR amplification with D2 primers under cycling conditions as described in Section 2.2.2 and evaluation by 2% agarose gel electrophoresis. In parallel, a CPV-specific ELISA assay was performed with rVP2 and cultured CPV as described previously, serving as the assay control. Buffer DL-extracted rVP2 content which was incubated with lambda exonuclease-digested (New England Biolabs, Ipswich, MA, USA) C1 ssDNA, served as the negative control for PCR, while synthesized ssDNA mix served as the positive control.

#### 2.4.3. Assessment of Virus-ssDNA Binding

Similarly to rVP2, intact CPV virus particles were coated onto a 96-well plate after diluting to 100 TCID_50_ per 100 µL in PBS. The assay steps, including PCR evaluation, were performed as stated above. To evaluate the binding properties of ssDNA to denatured virus particles, heat-treatment (95 °C for 10 min, then RT for 10 min) of intact virus particles was performed before adjusting to 100 TCID_50_ equivalent virus content per 100 µL for coating the plate.

For inducing denaturation by chemical treatment, peracetic acid (PAA) solution (Wolfasteril, Kesla, Bitterfeld-Wolfen, Germany) with a final concentration of 5% *v*/*v* and its 10-fold serial dilutions up to 0.005% *v*/*v* were added to the virus suspensions. The PAA reaction was performed for 15 min in a 1.5 mL tube with gentle shaking at RT, followed by resting the tubes for 10 min inside a laminar flow cabinet with the lid opened. The virus content was then diluted in PBS supplemented with 3 mM MgCl_2_ to the desired concentration (100 TCID_50_) for coating. To evaluate the effect of physical and chemical treatment on the efficiency of virus coating on the well surface, Bradford protein assay (ThermoFisher, MA, USA) was performed with the treated virus particles according to the manufacturer’s protocol. To evaluate the cross-binding activity of the aptamer candidates to related viruses other than CPV, as well as the expression host of the recombinant protein, both intact and heat-treated virus particles of PPV and FCV, as well as *E. coli*, were assessed for binding using the same protocol as stated above.

#### 2.4.4. Assessment of CPV Neutralization by ssDNA

To evaluate the effect of ssDNA binding to CPV on infecting host cells, ssDNA contents (10, 40, 80, and 160 pmol) were separately pre-incubated with 100 TCID_50_ virus suspensions in DMEM supplemented with 1× PBS and 3 mM MgCl_2_ for 2 h at two temperature conditions- room temperature (RT) or 37 °C. Next, 100 µL of each mixture was added to an equal volume of CRFK cells in DMEM media, which had been seeded at a concentration of 10^4^ cells/mL in a 96-well microtiter plate and supplemented with 3 mM MgCl_2_ and 10% FBS. The plate was then incubated for 5 to 6 days at 37 °C in a 5% CO_2_ environment. As a positive control for virus neutralization, antibody-positive serum was diluted 1:5 in PBS and incubated with 100 TCID_50_ virus particles at 37 °C for 1 h before being added to the CRFK cells as described above. For indirect immunofluorescence assessment of CPV-2 cytopathic effects (CPE), cells were fixed on the plates using a mixture of cold acetone and methanol, and CPV-specific monoclonal antibodies [26] were applied as previously described [21].

### 2.5. In Silico Binding Model and Data Analysis

To predict the binding affinity of aptamer candidates to the rVP2 protein and VP2 capsid of CPV, respective structures were obtained from the modelling servers and Protein Data Bank (PDB). An exemplary aptamer candidate sequence was first submitted to the RNAfold server [27] to predict its secondary structure, with energy parameters calculated for 25 °C. The secondary structure conformation was used to construct a 3D structure in RNAComposer [28]. The resulting PDB structure was then manually edited to replace uracil (U) residues with thymine (T) using BIOVIA Discovery Studio software v4.1 (Dassault Systèmes, San Diego, CA, USA).

The native CPV monomeric VP2 unit of the capsid protein was obtained from the PDB file (PDB ID: 4DPV), while a template-free ab initio model for rVP2 was constructed using DMPfold [29], which predicts inter-atomic distance bounds, torsion angles, and hydrogen bonds to build such models. The structures were refined using the YASARA energy minimization program [30]. Docking of the modeled aptamer candidate to the protein models was performed using the HDOCK server [31].

Enrichment analysis of sequences was conducted from NGS data to calculate the percentage frequency of each individual sequence. After generating a list of all unique sequences and counting the occurrence with the shell command “uniq-c” a graphical representation of enrichment was generated using the fastaptamer2 server [32]. Data analysis tests were performed to compare between and among datasets where appropriate, and the distribution of data was analyzed by using Prism v8 (GraphPad Software). A *p* value ≤ 0.05 was considered statistically significant.

## 3. Results

### 3.1. SELEX Procedure Results in Enriched ssDNA Sequences Against rVP2

An automated selection for 12 rounds was performed, and PCR products were obtained from each round. Next-generation sequencing (NGS) revealed the enrichment trend of unique ssDNA sequences with potentially high binding affinity to the rVP2 protein after the mid-round (C6) and final round (C12) selections, compared to the initial amplification which theoretically represents unbiased enrichment. The sequence 12-1 exhibited the highest abundance, covering 8.65% of the total sequences (only the first 500 sequences by frequency rank as given in Supplementary Data were considered) in the final round, while sequence 6-1 showed a maximum frequency of 2.14% in the mid-round selection (Table 1 and Figure S1). Control enrichment against Streptavidin showed approximately 23% maximum coverage for an enriched sequence and no homology was found to the maximum enriched sequences of the C6 and C12 selection rounds against rVP2 upon sequence alignment.

### 3.2. rVP2 Binds to Individually Enriched ssDNA Sequences but Lacks Antibody Binding Sites

**Figure 1:**
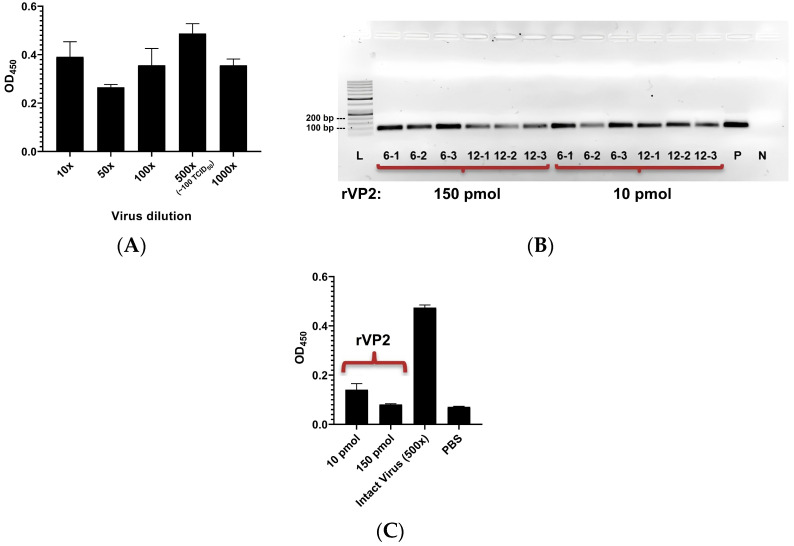
Establishment of rVP2-ssDNA binding assay. (**A**) OD_450nm_ NM measurement of antibody binding activity for different concentration of CPV; a 500× dilution of the stock CPV culture corresponds to approximately 100 TCID_50_ virus particles. (**B**) AGE (4% gel) visualization of PCR products derived from rVP2 bound ssDNA; L = ladder (50 bp), P = positive (PCR) control (synthesized ssDNA blend), N = negative control (extracted rVP2 content incubated with C1 ssDNA), (**C**) OD_450nm_ measurement of antibody binding activity (mean ± SD) for rVP2 (10 pmol: 0.14 ± 0.03, 150 pmol: 0.08 ± 0.003), 500× diluted stock equivalent to 100 TCID_50_ CPV particle (0.47 ± 0.01) and PBS (0.07 ± 0.002). The test was performed in triplicate.

For CPV, approximately 100 TCID_50_ virus particles equivalent dilution of stock CPV was found to be the best binder in terms of OD_450nm_ measurement in a CPV-specific ELISA assay (Figure 1A, One-way ANOVA: *p* < 0.01). Therefore, 10 pmol of rVP2 that measures roughly to 100 TCID_50_ weighted virus particles, and a higher concentration of rVP2 (150 pmol) were tested in 96-well plates.

The top three most enriched sequences from cycles C6 (6-1, 6-2, and 6-3) and C12 (12-1, 12-2, and 12-3) were evaluated for their binding to rVP2 (Table 1). Following incubation and subsequent extraction, PCR analysis confirmed binding of all six tested sequences to rVP2 at both concentrations with no apparent advantage of using higher concentration, while ssDNA from C1 did not yield detectable bands on agarose gel electrophoresis after PCR (Figure 1B).

Simultaneously, standard CPV-specific ELISA was conducted on rVP2 as an assay control, revealing significantly (*p* < 0.0001) lower binding of rVP2 (10 pmol) to CPV-specific monoclonal antibodies compared to equimolar (100 TCID_50_) intact virus particles (comparison of OD_450nm_ values, Figure 1C).

### 3.3. ssDNA Aptamer Candidates Binds to Denatured but Not to Intact CPV

**Figure 2:**
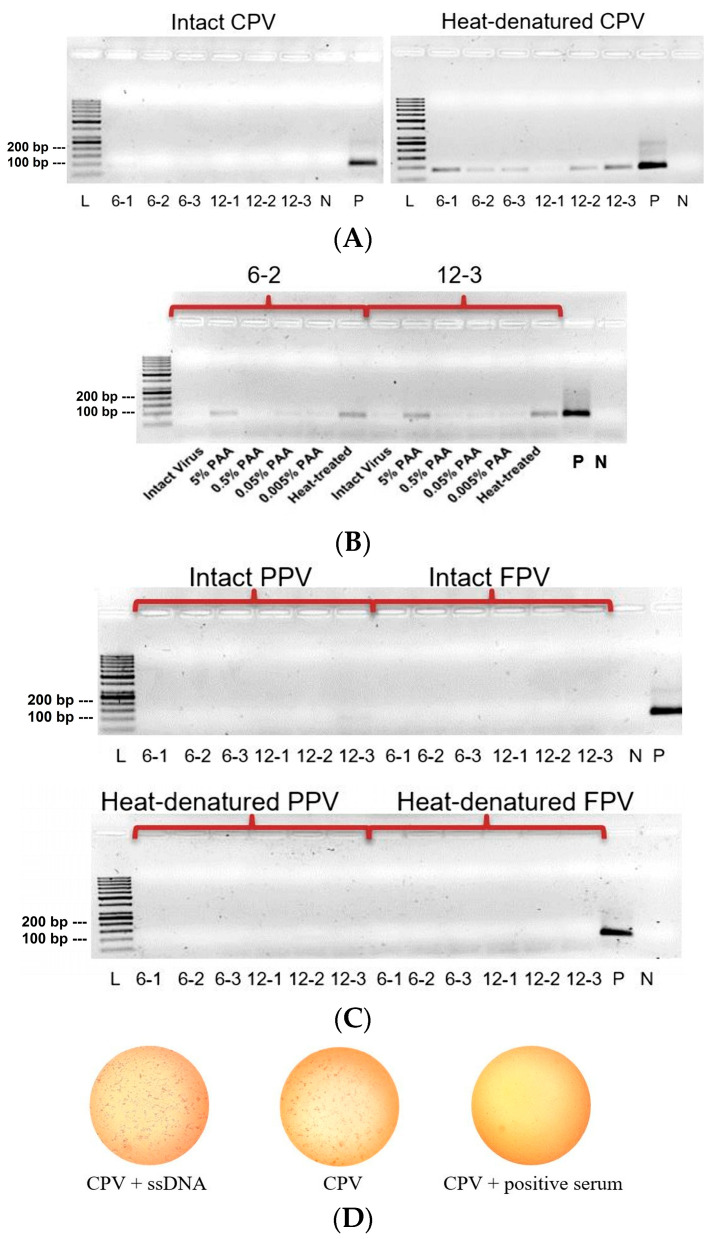
Evaluation of virus binding to ssDNA aptamer candidates. (**A**) CPV: intact and denatured forms after heat-treatment. (**B**) CPV: PAA treated in different concentrations, intact virus as negative control and heat-treated virus as assay positive control. (**C**) PPV and FPV: both intact and heat-treated forms. L = ladder (50 bp), L = ladder (50 bp), P = positive (PCR) control (synthesized ssDNA blend), N = negative control (extracted rVP2 content incubated with C1 ssDNA). (**D**) Cytopathic effects (CPE) characterised by agglutination of CRFK cells after CPV infection as visualised by immunofluorescence tests for CPV with ssDNA (CPE), CPV without ssDNA (CPE) and CPV with antibody positive serum (no CPE) and uniform cell growth. The tests were performed for all the six test sequences in duplicate.

In the next step, instead of rVP2, 100 TCID_50_ of CPV was coated in intact and heat-treated forms (95 °C for 10 min, followed by RT for 10 min). It was observed that all six enriched sequences exhibited bands of very low intensity or no bands to intact viruses. However, incubation of the test sequences with heat-treated virus resulted in detectable PCR bands except for 12-1 (less-intensive band), suggesting higher affinity for the denatured capsid (Figure 2A). Moreover, based on band intensities for heat-treated virus and comparative trend of frequencies at 6th and 12th round of selection (Table 1), two promising candidates (6-2 and 12-3) from each selection round were chosen for further testing against CPV treated with a range of PAA concentrations (from 5% *v*/*v* down to 0.005% *v*/*v*). Interestingly, similar to the heat-treated virus, bands were obtained for virus treated with a high concentration of PAA (5%), but faint or no bands were observed with lower concentrations (Figure 2B). However, this binding tendency was likely specific for CPV, as neither of the closely related viruses FPV and PPV (both intact and heat-treated forms, Figure 2C), nor *E. coli* (the host for recombinant protein expression), showed comparable band intensities for any of these six sequences. Furthermore, the ssDNA test sequences did not exhibit any neutralization effect on the virus when intact virus was incubated with the sequences followed by cell culture and indirect immunofluorescence assessment (Figure 2D).

### 3.4. ssDNA Binding to Virus Is Not Affected by Coating Efficiency Following Treatment

**Figure 3:**
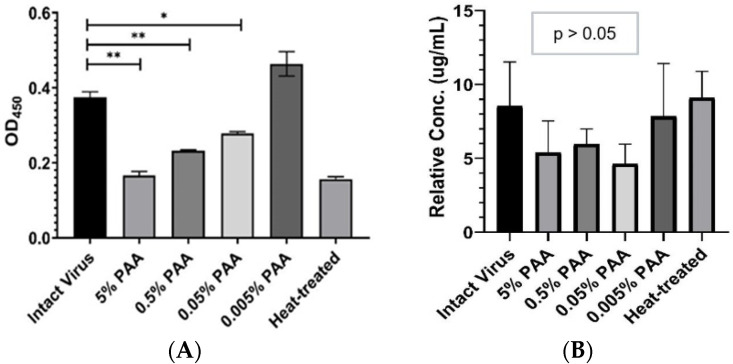
Evaluation of denaturing treatment effect on CPV. (**A**) Trend of antibody binding ability after treatment as indicated by OD_450nm_ measurement. * *p* < 0.05, ** *p* < 0.005. (**B**) Plate coating concentration after treatment.

Similarly to the rVP2 protein, the heat- and PAA-treated virus significantly lacked antibody binding ability as shown in Figure 3A in terms of OD_450nm_ measurement (mean ± SD) were: intact virus (0.37 ± 0.01), heat-treated virus (0.15 ± 0.007), and treated virus with 5% PAA (0.16 ± 0.01), 0.5% PAA (0.23 ± 0.002), 0.05% PAA (0.28 ± 0.005) and 0.005% PAA (0.46 ± 0.03). This indicates deformation of antigen recognition sites on the viral capsid. However, this lack of antibody binding activity was unlikely to be associated with the relative coated protein concentration with or without any treatment (One-way ANOVA, *p* > 0.5) as shown in Figure 3B. The relative concentration (mean ± SD) in µg/mL was: intact virus (8.54 ± 2.98), heat-treated virus (9.12 ± 1.77), and treated virus with 5% PAA (5.39 ± 2.14), 0.5% PAA (5.96 ± 1.03), 0.05% PAA (4.63 ± 1.34) and 0.005% PAA (7.85 ± 3.57). This suggests that ssDNA binding to heat- and PAA-treated virus coated on the plate surface is unlikely to be affected by the coating efficiency.

### 3.5. Variable Monomeric Interaction of VP2 Protein with ssDNA Aptamer Candidate

**Figure 4:**
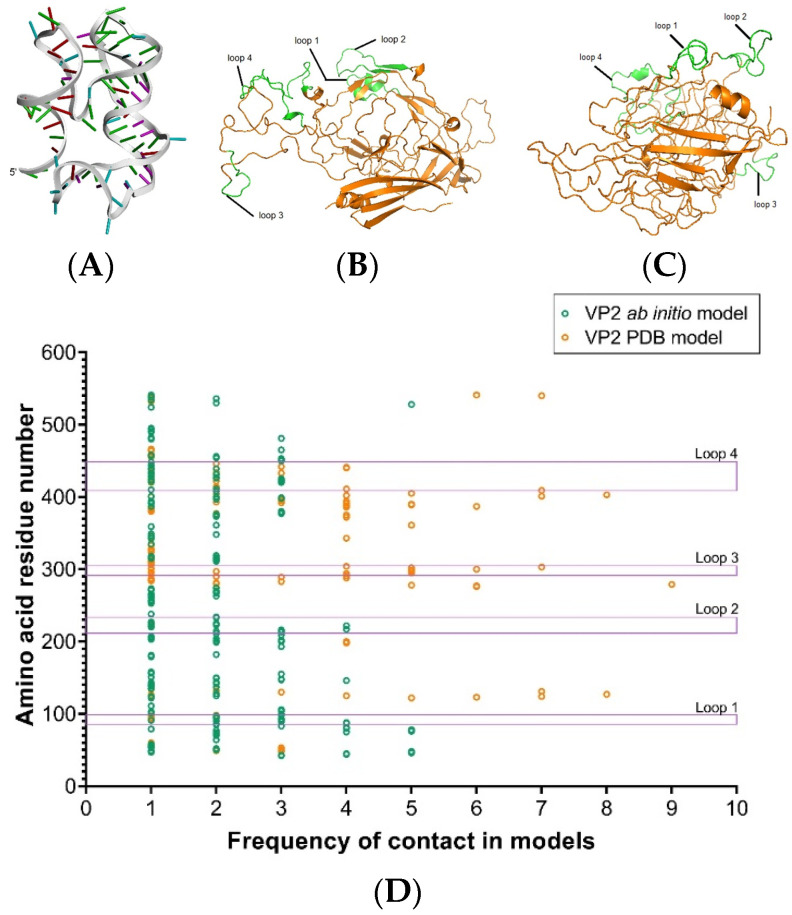
In silico prediction of ssDNA and VP2 structures, and their modes of interaction. (**A**) Predicted structure of ssDNA 12-3. (**B**) Monomeric unit of the CPV capsid derived from PDB entry 4DPV. (**C**) Template-free ab initio construct of the VP2 monomeric unit, with loop amino acid positions indicated in green: loop 1 (positions 84–99), loop 2 (positions 216–235), loop 3 (positions 295–306), and loop 4 (positions 409–444). (**D**) Frequency mapping of ssDNA 12-3 contact points with the amino acid residues of the 4DPV (PDB model) monomeric unit and the ab initio monomeric structure for the top 10 binding models. Note that loop amino acid positions are not drawn to scale.

To deduce the ssDNA binding trend to rVP2, a template-free in silico structure of VP2 was determined to compare the binding trend with the available structural unit (PDB ID: 4DPV). This was done under the assumption that the denatured capsid of CPV may unfold into a monomeric unit of the VP2 protein upon treatment and/or refold into structures not akin to the native assembly of VP2 (i.e., outside the host cell) once the treatment conditions are withdrawn. As an example interaction, the secondary structure of ssDNA aptamer candidate number 12-3 was first constructed with a minimum free energy of −29.04 kcal/mol (Figure S2). This structure was then used to obtain the 3D conformation of the ssDNA (Figure 4A). The docked complexes obtained with 12-3 for both the resolved monomeric unit and for the template-free ab initio model showed structural variation (Figure 4B,C) as well as distinct binding preferences. HDOCK returned 10 possible binding modes ranked by docking score. The ab initio VP2 structure was predicted to have more variable binding sites for the tested ssDNA than the resolved VP2 monomeric unit obtained from the PDB server. Loop and near-loop residues, particularly those of loop 3 (residues 295–306) along with residues 122–131 were predicted to be frequent binders in the docked complexes for the PDB model, while ab initio model did not seem to have such frequent binding regions (Figure 4D and Figure S3).

## 4. Discussion

This study evaluated whether nucleic acid aptamers can differentiate between intact virus particles and their denatured forms resulting from physical or chemical disinfection treatments. For this purpose, ssDNA binders to the recombinant VP2 capsid protein of CPV were selected through SELEX. The six most enriched sequences after the mid- and final SELEX rounds were then assayed individually to evaluate their binding to both rVP2 and cultured CPV. Assays were also performed to test the binding of these sequences to heat- and PAA-treated CPV, as well as for cross-binding to viruses closely related to CPV, such as PPV and FPV. Our observations suggest that the tested aptamer candidates demonstrated preferential binding to heat- or chemically treated CPV particles compared to intact ones, indicating their capability to distinguish between the two states. However, AGE visualization provides only limited information on the difference in binding affinity among the tested sequences; we assume that the enriched sequences may share common motifs that determine the structural orientation and binding interface.

CPV belongs to the non-enveloped *Parvoviridae*. Due to its small, compact and simple structure with an icosahedral capsid (~25 nm in diameter) and hydrophilic properties, CPV is more difficult to inactivate with common chemical disinfectants than lipophilic non-enveloped viruses. However, oxidizers such as hydrogen peroxide and peracetic acid, chlorine releasers such as sodium hypochlorite, and aldehydes have been proven effective [33]. This is why CPV was selected as the non-enveloped representative in this study for aptamer candidate selection and binding evaluation following disinfection treatment. On the other hand, enveloped viruses are generally more prone to inactivation by common chemical disinfectants, making the choice of disinfectant more straightforward. Nevertheless, future studies could assess enveloped viruses can also be assessed for disinfectant-mediated inactivation using the aptamer recognition approach presented in this study.

CPV is assembled from 60 copies of capsid proteins, VP1 and VP2, with VP2 constituting the major capsid protein, making up around 90% of the CPV capsid. VP2, when produced as a recombinant protein in bacterial or eukaryotic hosts, is known to self-assemble into virus-like particles (VLPs) [34,35], which are essentially viruses with conformational arrangements almost identical to intact CPV virions but without genomic content. VLPs are of great interest for developing biomimetic platforms in diagnostic and therapeutic applications, as well as in vaccine development, as discussed elsewhere [36]. VLPs support the idea that for an emerging virus with no established cell-culture method, recombinant capsid proteins that self-assemble into VLPs after being produced from genomic information might facilitate a faster and cheaper way to test disinfection measures, bypassing the need for purified non-enveloped viruses or their handling in BSL labs. However, recombinant protein production in complex (e.g., eukaryotic) hosts is often costly, limited in production capacity, and complicated in downstream purification, compared to simple bacterial hosts like *E. coli* [37,38]. Hence, rVP2 was produced in a bacterial host (*E. coli*) to simplify and expedite the production of recombinant capsid protein in this study.

In the next step, an automated SELEX module was used to screen potential ssDNA aptamers from a random library for rVP2, facilitating the selection cycles without direct manual intervention. Major advantages of the automated SELEX procedure include speed, accuracy, and reduced variability in repetitive steps [39,40]. In less than 60 h, we generated 12 rounds of SELEX products for downstream enrichment analysis of the sequences (Figure S1). However, in general, the cost and expertise associated with infrastructure and maintenance may limit its utility to specialized laboratories for automated aptamer candidate selection. Both RNA and ssDNA aptamers can form secondary and tertiary structures and have emerged for diagnostic and therapeutic applications due to their specific recognition of biomolecular conformations [41]. All six tested ssDNA sequences in the study share the common feature of high GC content, which tends to form strong hydrogen bonds in stable secondary structures (Table 1).

Although numerous reports on aptamers demonstrate their potential in diagnostic and therapeutic applications for viral pathogens, studies reporting aptamer efficiency in differentiating inactivated viruses from intact viruses remain scarce. A key factor in this aspect is the use of denatured virus following the target disinfection treatment as a control. Aptamers against CPV capsid protein VP2 have been previously reported with encouraging specificity for diagnostic purposes [42]; however, aptamer behavior toward inactivated CPV was not assessed. Another report suggested counter SELEX selection of aptamer candidates against UV-irradiated non-infectious pseudoviruses of SARS-CoV and H5N1 (both enveloped) in addition to regular selection rounds against the infectious form [43]. To our knowledge, we evaluated for the first time the differential aptamer-binding between intact and heat or PAA-treated CPV. We standardized the evaluation system by incorporating cross-binding specificity checks of the aptamer candidates to CPV-related viruses such as PPV and FPV, as well as by assessing their neutralization ability to prevent infection of the cell line (Figure 2). The lack of neutralization of intact CPV by the selected aptamers indicates that viral infectivity is unaffected by aptamer binding and hence minimizes the risk of false negatives when assessing disinfectant-treated virus.

Interestingly, the tested aptamer candidates showed higher binding intensity to chemical and heat-treated virus rather than intact ones. This suggests that aptamer binding reflects denatured capsid and that recognition of inactivated CPV may vary depending on whether the inactivation method perturbs capsid conformation. In consequence, an attenuated but not inactivated CPV particle is also unlikely to be recognized by the selected aptamers because its capsid morphology remains intact and closely resembles that of wild-type CPV. Therefore, in this study, aptamer binding can be considered markers of structural damage rather than surrogates of infectivity loss, and additional studies would be imperative to establish the relationship between the concentration of intact or denatured virus and the relative infectivity level in cell culture. Furthermore, the binding trend also suggests a higher affinity of the aptamer candidates for denatured CPV generated by heat or PAA disinfectant treatment rather than for intact virus. Due to significantly less anti-CPV antibody binding against rVP2 protein compared to that against intact CPV, it can be speculated that the rVP2 used in the SELEX selection process, against which aptamers were screened, may not be self-assembled into CPV VLPs. This is plausible, as for the self-assembly of VP2 monomeric units of a eukaryotic virus like CPV, capsid elements need to undergo post-translational modification following synthesis in host cells or eukaryotic vectors [44,45,46], and may require co-expression of modifier proteins if expressed in a prokaryotic host like *E. coli* for native assembly of monomeric units [35,47]. It is also possible that upon treatment with heat or a denaturing agent like PAA, the native structure of CPV unfolds into monomeric units of VP2, where candidate aptamer sequences have preferable binding sites as in unassembled rVP2, which are not accessible in the assembled capsid. The tested aptamer candidates were unable to neutralize intact CPV against infection in CRFK cells, suggesting non-binding to host attachment sites of the virus. Furthermore, in in silico analysis of the binding trend, we evaluated both possible conformations: a monomeric unit of native VP2 (PDB model) and a template-free ab initio conformation of VP2 built using only sequence information. The structural differences between the two conformations are also reflected in the binding preferences. We used a test aptamer candidate (12-3) to show the 10 most preferable binding models for the candidate. Interestingly, when the PDB model was used, contact sites involving residues of loop 3 and roughly within residues 122–131 were predicted to be highly frequent among the possible binding models (Figure 4D and Figure S3). However, in self-assembled parvoviruses, the loop residues are likely pre-occupied in intermolecular bonds among monomeric units to form the capsid structure [48,49].

The main goal of the study was to assess whether an aptamer-based assay can differentiate between intact virus particles and disinfectant treated ones. In this context, the evaluation was performed qualitatively only (i.e., assessment by PCR). We used a simple initial assessment to test the ability of aptamer as a marker in differentiating the integrity of disinfectant treated virus from the intact ones which can be performed in regular laboratories without high-end equipment. However, it is important to quantify the aptamer binding metrics by performing quantitative assays such as quantitative PCR, enzyme-linked oligonucleotide assay (ELONA) and surface plasmon resonance (SPR) to measure the affinity of binding between aptamer and target molecule as well as to test specificity. Future studies evaluating the applicability of an aptamer candidate as an alternative marker for assessing disinfection efficacy against a target virus should consider this to establish a quantifiable measurement system for comparison with cell culture-based conventional methods. Furthermore, extensive cross-reactivity testing of aptamer candidates across multiple virus families would be necessary to better understand the specificity of aptamer candidates and to avoid misinterpretation of efficacy. While standardized infectivity assays remain necessary for regulatory validation of disinfectant efficacy, comparative studies that quantitatively correlate viral particle integrity with infectivity levels could support the development of alternative approaches to infectivity testing, potentially reducing reliance on time-consuming and labor-intensive culture-based methods.

Furthermore, despite the *E. coli* expression system of being a relatively simple and widely accepted approach for recombinant protein production, it is crucial for the capsid protein to properly fold to generate VLPs with correct conformational arrangements. Therefore, future studies should consider eukaryotic expression systems of recombinant capsid protein, preferentially systems that facilitate post-translational modifications of the expressed protein. The production of the VLPs should be accompanied by evaluation for folding with transmission electron microscopy. As an alternative, SELEX selection of candidate aptamers against intact but attenuated CPV can be performed. Additionally, a counter selection round against target disinfectant-treated denatured virus in SELEX can be performed after positive selection to rule out cross-binding nucleotide sequences. Lastly, a representative of non-enveloped viruses- CPV- was preferentially selected in this study due to its well-documented resistance to chemical inactivation. However, variations among non-enveloped viruses can influence disinfection outcomes as differences in capsid conformation and physicochemical properties affect susceptibility to treatment. Structural differences may also impact aptamer discovery as variations in capsid proteins across virus families or among strains can alter binding affinity and specificity. Disinfectant-induced conformational changes may further modify these epitopes, influencing whether aptamers can still differentiate intact from denatured particles. Therefore, evaluations of disinfectant efficacy and aptamer-based detection potential should be performed in a virus-specific manner. In addition, different disinfectants may affect the viral particles differently based on the type of active component, and therefore their impact on capsid integrity and aptamer binding should be evaluated independently. Nevertheless, despite the limitations, this study demonstrates that an aptamer-based assay can differentiate between intact and disinfection-treated disintegrated virus and hence convey the potential to develop a quantitative molecular assay for evaluating the virucidal activity of test disinfectants.

## 5. Conclusions

Despite certain limitations, this study demonstrates that an aptamer-based assay can effectively distinguish between intact and disinfectant-treated viruses. The qualitative binding assay can act as a rapid and feasible primary evaluation method to test a disinfectant. The outcomes in the study highlight its potential for developing a quantitative molecular assay to evaluate the virucidal effectiveness of disinfectants. Future studies involving quantitative analysis will be imperative to understand binding affinity and specificities of aptamer candidates to intact and disinfectant-treated viruses.

## Figures and Tables

**Table 1:** Selected ssDNA aptamer candidates from SELEX rounds C6 and C12, showing sequence information and enrichment frequencies calculated from the top 500 sequences by rank.

SELEX Round	Rank	Sequence	Freq. C6 (%)	Freq. C12 (%)
C6	1	5′ D2Fw-AGGGAGGGGATCGGGTGGGGGGACTGCATCCATCTCTATT-D2Rv 3′	3236 (2.14)	6588 (1.84)
	2	5′ D2Fw-TGGGTGGGAGGGGCTCTCGGGGGGTCTTCCTAGGTTTGGT-D2Rv 3′	2664 (1.76)	9718 (2.72)
	3	5′ D2Fw-TGGGCGGGAGGGGATTCGGGGGGCACCGTTTTTTTACGGT-D2Rv 3′	2173 (1.44)	9307 (2.6)
C12	1	5′ D2Fw-TGTGGAGGCGGGCTGGGGAGGCGGGGGAGCTACTTCATCG-D2Rv 3′	579 (0.38)	38,597 (8.65)
	2	5′ D2Fw-GAGTGGCGGAGGGTGGGGAGGTGGGGGCCTGACTGGGCCT-D2Rv 3′	1056 (0.7)	33,527 (7.06)
	3	5′ D2Fw-GGTGGGCGGTGGGGGGGTCGCCGGTGGGCCCTCTTACGAT-D2Rv 3′	1939 (1.28)	30,560 (6.85)

## Data Availability

Data is contained within the article or Supplementary Materials.

## Supplementary Materials

The following supporting information can be downloaded at: https://www.mdpi.com/article/10.3390/v17101309/s1, Supplementary Data: 500 nucleotide base sequences by frequency rank. Supplementary Information: Table S1: Selected ssDNA aptamer candidates among the first 500 enriched sequences by rank and predicted sequence motifs for the selected rounds; Figure S1: SELEX selection profile; Figure S2: Predicted minimum free energy (MFE) secondary structures of aptamer candidates; Figure S3: In silico docking models for VP2 (golden) protein.

## Abbreviations

The following abbreviations are used in this manuscript:

SELEX	Systematic Evolution of Ligands by Exponential Enrichment
CPV	Canine Parvovirus
PPV	Porcine Parvovirus
FPV	Feline Panleukopenia Virus
rVP2	Recombinant VP2
VLP	Virus-Like Particle
PAA	PerAcetic Acid
ssDNA	Single Stranded DeoxyriboNucleic Acid
TCID_50_	50% Tissue Culture Infectious Dose
