# Supplementary material for: Exploring an Aptamer-Based Approach to Assess Canine Parvovirus Integrity After Disinfection Treatment
Source: Viruses. 2025 Sep 27;17(10):1309. doi: 10.3390/v17101309 (PMC12568004; doi:10.3390/v17101309)
Supplement: Supplementary file 1 [file viruses-17-01309-s001.zip › Supplementary Information_revised.pdf]

Table S1: Selected ssDNA aptamer candidates among the first 500 enriched sequences by rank and predicted sequence motifs for the selected rounds

SELEX round	Rank	Sequence
C6	1	5’D2Fw-AGGGAGGGGATCGGGTGGGGGGACTGCATCCATCTCTATT-D2Rv 3’
	2	5’ D2Fw-TGGGTGGGAGGGGCTCTCGGGGGGTCTTCCTAGGTTTGGT-D2Rv 3’
	3	5’ D2Fw-TGGGCGGGAGGGGATTCGGGGGGCACCGTTTTTTTACGGT-D2Rv 3’
	Motif logo & number of sequence sites	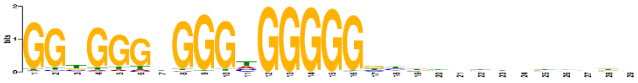 (458)
C12	1	5’ D2Fw-TGTGGAGGCGGGCTGGGGAGGCGGGGGAGCTACTTCATCG-D2Rv 3’
	2	5’ D2Fw-GAGTGGCGGAGGGTGGGGAGGTGGGGGCCTGACTGGGCCT-D2Rv 3’
	3	5’ D2Fw-GGTGGGCGGTGGGGGGGTCGCCGGTGGGCCCTCTTACGAT-D2Rv 3’
	Motif logo & number of sequence sites	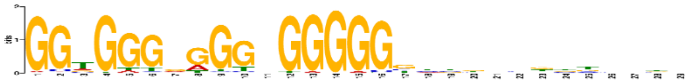 (487) 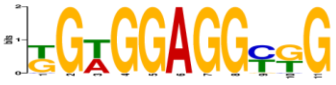 (37) 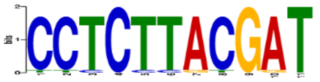 (20) 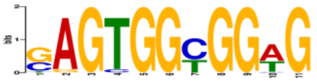 (32)

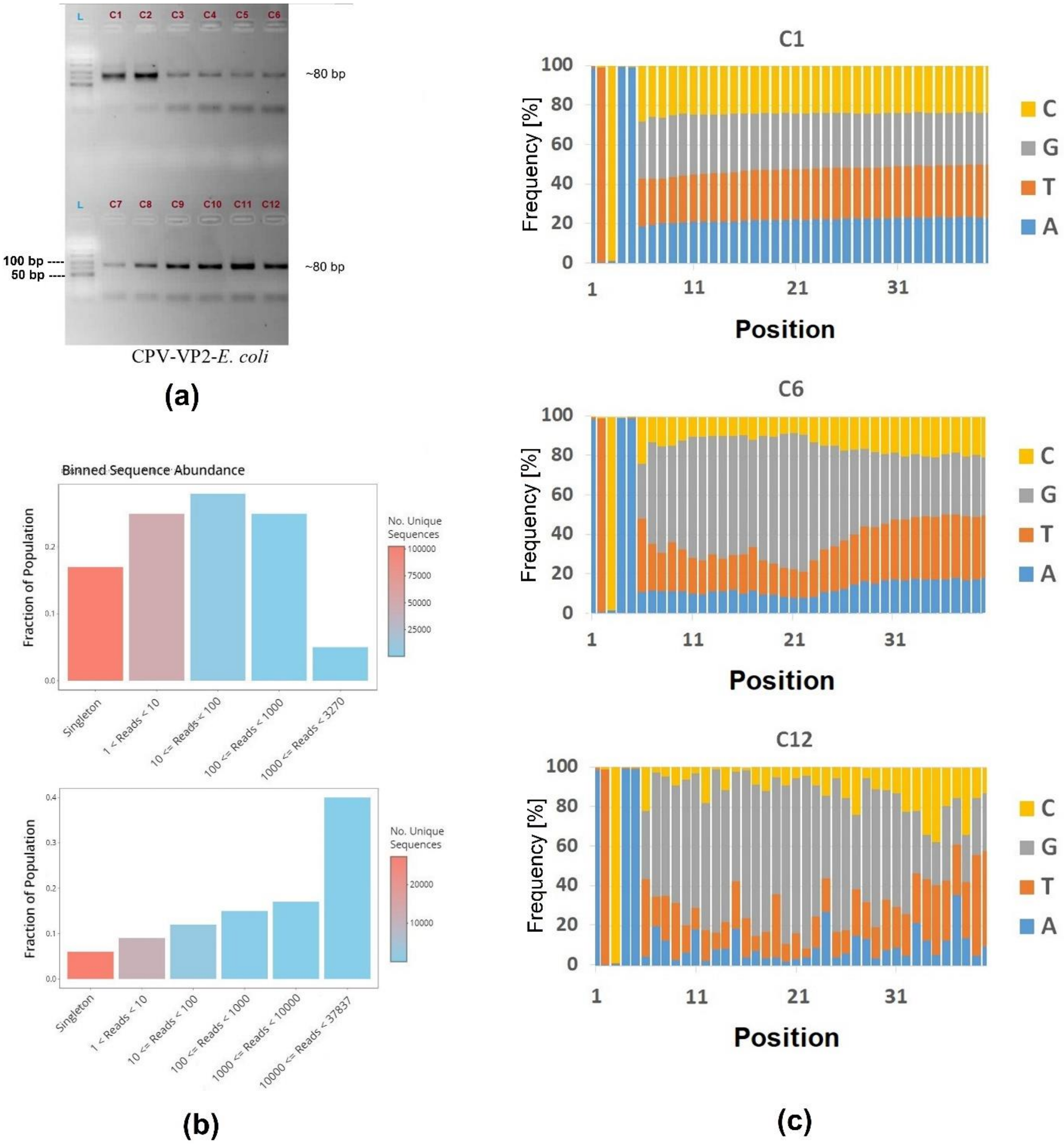

Figure S1: SELEX selection profile. (a) AGE of enriched DNA (~80 bp) after each round of selection (C1-C12); L = ladder (25 bp), (b) enrichment of unique sequences after C6 (top) and C12 (bottom) rounds, and (c) frequency of bases in random positions after C1 C6 and C12 rounds.

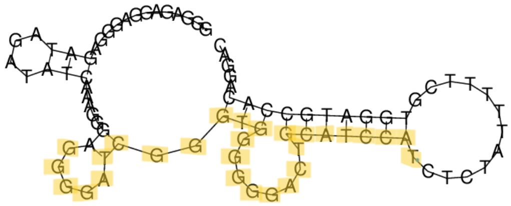

Candidate no.: 6-1
MFE (RNA): -28.61 kcal/mol
MFE (DNA): -9.69 kcal/mol

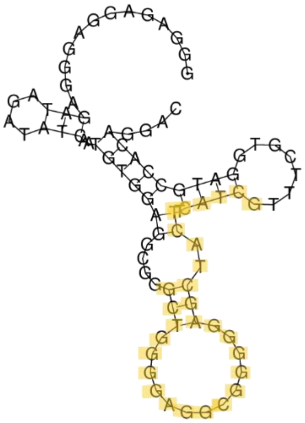

Candidate no.: 12-1
MFE (RNA): -25.75 kcal/mol
MFE (DNA): -8.12 kcal/mol

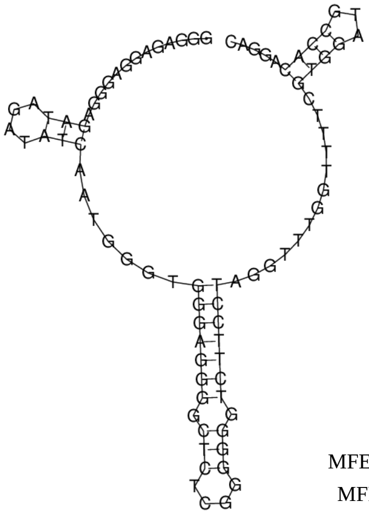

Candidate no.: 6-2
MFE (RNA): -27.92 kcal/mol
MFE (DNA): -7.22 kcal/mol

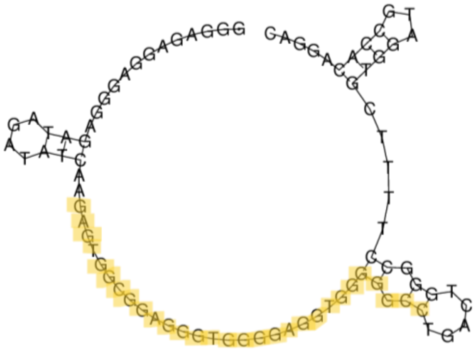

Candidate no.: 12-2
MFE (RNA): -27.70 kcal/mol
MFE (DNA): -9.45 kcal/mol

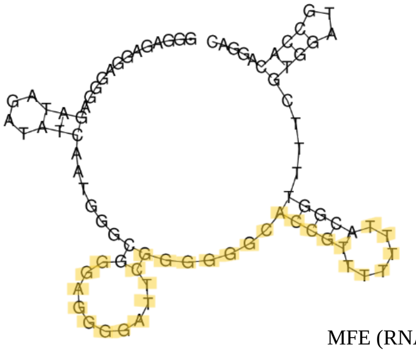

Candidate no.: 6-3
MFE (RNA): -25.02 kcal/mol
MFE (DNA): -9.85 kcal/mol

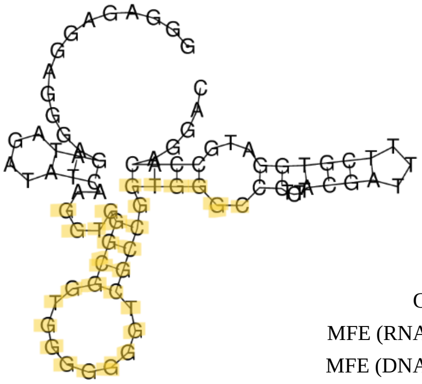

Candidate no.: 12-3
MFE (RNA): -29.04 kcal/mol
MFE (DNA): -12.84 kcal/mol

Figure S2: Predicted minimum free energy (MFE) secondary structures of aptamer candidates. MFE values were calculated using RNA or DNA parameters. The bases in the motif region commonly shared by the candidates (underlined red in Table S1) is highlighted yellow.

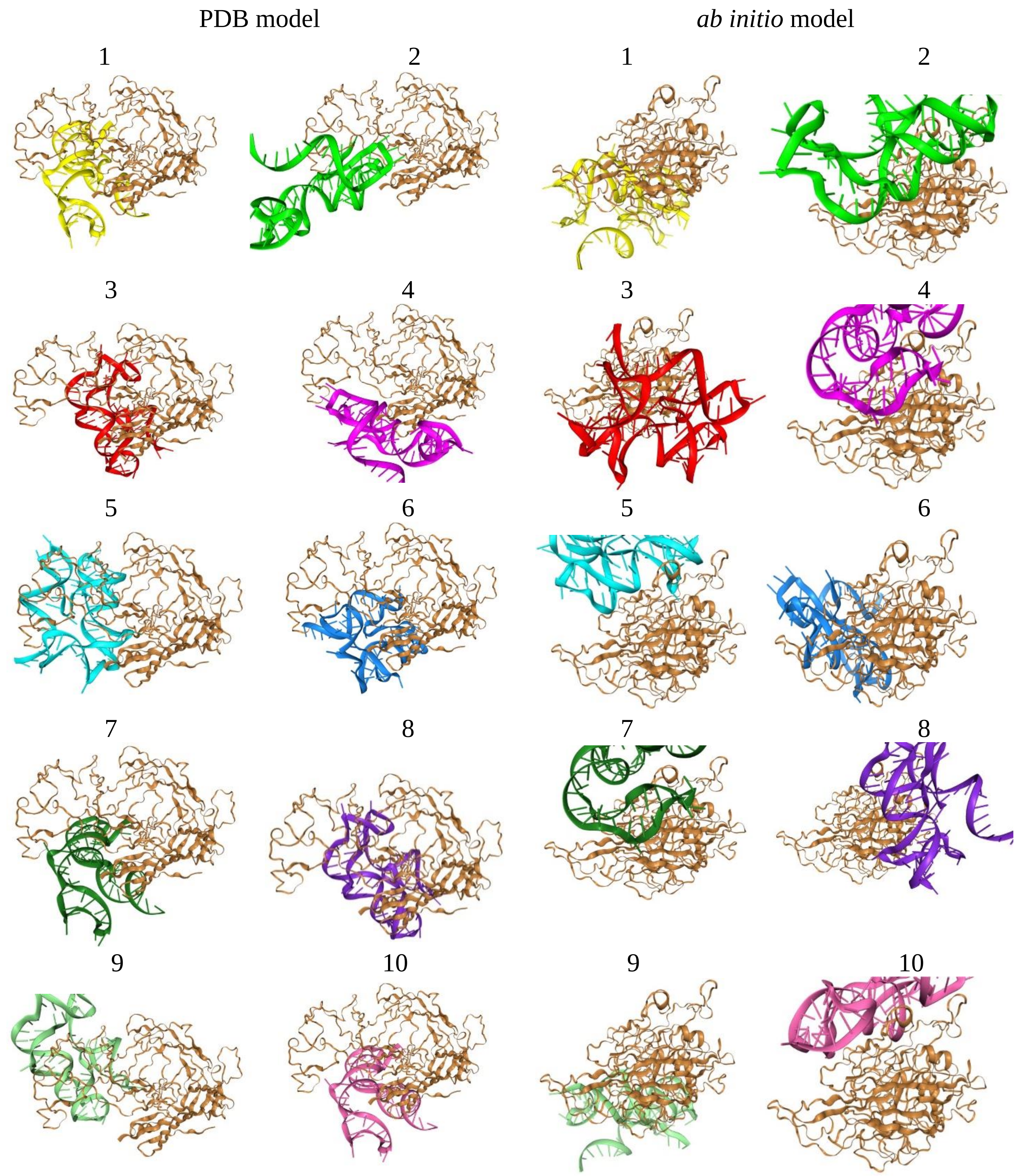

Figure S3: *In silico* docking models for VP2 (golden) protein
